# Self-Adaptive Polymer Fabry–Pérot Thermometer for High-Sensitivity and Wide-Linear-Range Sensing

**DOI:** 10.3390/bios15090602

**Published:** 2025-09-12

**Authors:** Yifan Cheng, Maolin Yu, Junjie Liu, Yingling Tan, Jinhui Chen

**Affiliations:** 1 School of Electronic Science and Engineering, Xiamen University, Xiamen 361005, China; 34320221150220@stu.xmu.edu.cn (Y.C.); 34320231150238@stu.xmu.edu.cn (M.Y.); 34320241150315@stu.xmu.edu.cn (J.L.); 2 Shenzhen Research Institute of Xiamen University, Shenzhen 518000, China

**Keywords:** fiber optic sensors, thermo-optic effect, cross-correlation algorithm, thermal detection

## Abstract

Fiber-optic temperature sensors with advantages such as simplicity, low cost, and high sensitivity have attracted increasing attention. In this work, we propose a self-adaptive polymer Fabry–Pérot interferometer (PFPI) sensor for ultrasensitive and wide-linear-range thermal sensing. This design achieves a temperature sensitivity of 0.95 nm/°C, representing an enhancement of two orders of magnitude compared to conventional fiber Bragg gratings. To address the challenge of spectral shifts exceeding the free spectral range due to the high sensitivity, a local cross-correlation algorithm is introduced for accurate wavelength tracking. We demonstrate ultrahigh-resolution (0.025 °C) scanning thermal field imaging and sensitive human physiological monitoring, including precise body temperature and respiratory rate detection. These results highlight the dual capability of our PFPI sensor for both microscopic thermal mapping and non-invasive healthcare applications.

## 1. Introduction

Accurate temperature measurement and thermal mapping hold profound significance across many scientific and industrial domains. In nuclear safety, real-time monitoring of reactors and water-cooled system temperatures is critical to prevent catastrophic failures and ensure operational integrity [1]. Meanwhile, in physiological research, the hypothalamus is the body’s central thermostat, necessitating advanced temperature monitoring techniques to elucidate its intricate thermoregulatory mechanisms [2]. In addition, the body temperature variation contains significant physiological information of the pathological status, and continuous long-term monitoring of physiological temperature using wearable epidermal sensors is attracting research interest for their great potential for diversified clinical purposes [3,4,5,6,7]. Beyond these applications, high-precision thermometry is indispensable in biomedicine [8,9,10,11], optoelectronic devices [12,13,14,15,16,17,18,19], livestock farming [20], and the aerospace industry [21,22]. For example, in tumor thermal ablation therapy [23], spatially resolved temperature control is indispensable—it is necessary to kill tumor cells while protecting surrounding healthy tissues. Furthermore, for intracellular chemical reaction processes [10], temperature is also a crucial monitoring parameter. These diverse applications underscore the urgent need for thermometers with high sensitivity, high spatial resolution, and high dynamic range.

Contemporary advanced temperature measurement has evolved significantly from traditional liquid-expansion thermometers to automated electrical and optical sensors [24,25,26,27,28], yet critical challenges persist. Thermistors and thermocouples remain widely used among electrical sensors due to their simplicity and scalability [29,30,31,32]. However, thermistors suffer from nonlinear response curves, while thermocouples exhibit limited robustness in harsh environments [33,34]. Infrared thermography, though non-contact, faces intrinsic trade-offs between spatial resolution and accuracy, restricting its use in precision applications [35]. In contrast, fiber-optic temperature sensors offer compelling advantages, including electromagnetic interference immunity and environmental stability [36,37,38]. In particular, fiber Bragg grating (FBG) sensors can achieve absolute thermometry without reference but are constrained by low temperature sensitivity (~10.5 pm/°C) [39,40,41]. Alternative designs leveraging interferometers [42,43] or tapered fibers similarly struggle with silica-material-limited sensitivity [36,41]. While cascaded/parallel interferometric structures exploit the Vernier effect to enhance sensitivity [44,45], their performance is prone to multi-parameter crosstalk and signal instability. Using liquid-crystal-filled fiber sensors demonstrates high temperature sensitivity, yet long-term reliability is compromised by inevitable fluid evaporation [46], and the microfluidic design adds to the increased sensor footprint. Recent advances in polydimethylsiloxane (PDMS)-based interferometric sensors have demonstrated exceptional sensitivity in pressure and temperature sensing, thanks to the high thermal expansion coefficient and thermo-optic coefficient [47,48,49]. For instance, Zhao et al. developed a PDMS-filled FPI hydraulic pressure sensor with a sensitivity of −7.35 nm/kPa, yet its performance was susceptible to temperature fluctuations [50]. To address this issue, Tang et al. proposed a thin-film PDMS-Fabry–Pérot-interferometer pressure sensor (29.64 nm/MPa sensitivity) combined with an FBG for temperature compensation, albeit at the cost of increased structural complexity [51]. Zhou et al. [48] demonstrated PDMS-coated Mach–Zehnder interferometer with a temperature sensitivity as high as 8.2 nm/°C.

**Figure 1:**
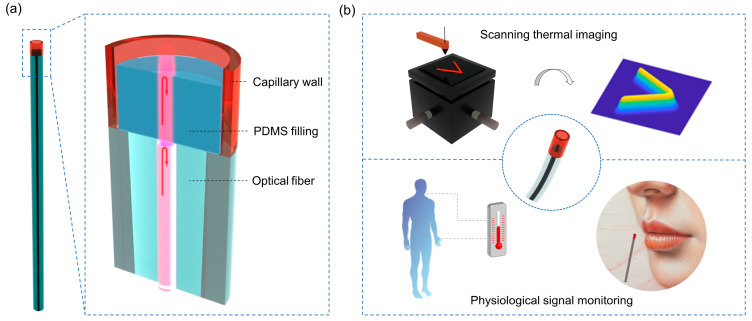
Self-adaptive polymer Fabry–Pérot interferometer (PFPI) and its thermal sensing applications: (**a**) Schematic diagram of the PFPI structure. The PFPI is composed of a single-mode optical fiber spliced with a glass microcapillary, and the hollow core region is filled with polydimethylsiloxane (PDMS). The light reflections at the interface of silica-PDMS and PDMS-air form a Fabry–Pérot interferometer (FPI). (**b**) Thermal sensing applications of the PFPI include scanning thermal imaging and human physiological signal monitoring.

In this work, we propose a self-adaptive polymer Fabry–Pérot interferometer (PFPI) for ultra-sensitive and wide linear range thermal sensing. The PFPI sensor is fabricated by filling PDMS into a section of microcapillary to form an optical cavity structure, resulting in higher mechanical stability and repeatability. The ultracompact PFPI (~100 μm) is self-formed by infilling polydimethylsiloxane (PDMS) into a glass microcapillary, spliced with a single-mode fiber (Figure 1a). We demonstrate theoretically and experimentally that PFPI achieves a temperature sensitivity of 0.95 nm/°C, mainly contributed by the thermal expansion effect of PDMS, which is two orders of magnitude higher than that of the conventional FBG sensors. We introduce the cross-correlation algorithm to decode the spectral shift in PFPI and avoid the track-loss issue beyond a free spectral range using the classical mode-shift method. Based on the proposed PFPI, we demonstrate the versatile applications of scanning thermal field imaging with an ultrahigh resolution of 0.025 °C and sensitive human physiological signal measurement, including body temperature and respiratory rate (Figure 1b). Our results demonstrate the versatile potential of PFPI sensor in the field of biomedical monitoring.

## 2. Materials and Methods

### 2.1. Preparation and Characterization of PFPI Sensors

Figure 1a illustrates the optical structures of the designed PFPI sensor, which is integrated onto a single-mode fiber endface. The infilled PDMS (Polydimethylsiloxane, Dow corning, Midland, MI, USA, DC184) in the hollow core region solidifies and naturally forms a cylinder-like structure under the constraints of the fiber end face and capillary wall. The Fabry–Pérot interferometer (FPI) is constructed by the light reflection at the interfaces of silica-PDMS and PDMS-air. The detailed preparation processes of the PFPI sensor are as follows. First, a fiber cleaver is used to cut the single-mode fiber (G.652D, Corning, Corning, NY, USA, diameter 125 μm) and glass microcapillary (TSP100170, Polymicro Technologies, Phoenix, AZ, USA, outer diameter 125 μm, inner diameter 75 μm), and the microcapillary is spliced with an optical fiber via the fusion splicer. The attached microcapillary is cut short, leaving a typical length of ~300 μm on the fiber endface. Then, the prepared uncured PDMS is permeated into the microcapillary, and the cured shape of the PDMS is constrained by the inner wall of the microcapillary. It is worth noting that the PDMS-filled length in the capillary primarily affects the free spectral range (FSR) of the device while having little impact on the temperature sensitivity. The typical filling length of PMDS in the experiment is around 150 μm. Such controlled cavity length is mainly based on considerations from several aspects. On one hand, an excessively small cavity length may lead to a decrease in the interference intensity contrast and the increased fabrication difficulty; on the other hand, an excessively large cavity length may cause greater optical loss. Notably, the PDMS-filled device is placed in a vacuum chamber to evacuate air and eliminate trapped bubbles. Finally, the PDMS is cured and solidified, and the PFPI sensor is obtained. We also fabricate conventional air-filled Fabry–Pérot interferometer (APFI) sensors of fiber-microcapillary-fiber structures. The microcapillary is cleaved under the optical microscope, such that its length can be precisely controlled. The sealing side with single-mode fiber is obtained by optimizing the splicing parameters.

For fiber-optic FPI sensors, the resonant wavelength satisfies the standing wave condition

(1) $$\lambda =\frac{2nl}{m}$$

where *λ* is the resonant wavelength, *n* is the refractive index of the cavity, *l* is the cavity length, and *m* is a positive integer. The analytical temperature sensitivity of FPI sensors can be written as:

(2) $$\frac{∆\lambda }{∆T}=\left(\frac{1}{l}\frac{∆l}{∆T}+\frac{1}{n}\frac{∆n}{∆T}\right)\lambda =(\alpha +\kappa )\lambda$$

where *α* and *κ* represent the thermal expansion coefficient and thermo-optic coefficient of the medium in the cavity, respectively. For PDMS material, the volume thermal expansion coefficient and thermo-optic coefficient are 9.6 × 10^−4^ °C^−1^ [52] and −4.5 × 10^−4^ °C^−1^ [53], respectively. Consequently, the predicted temperature sensitivity of the PFPI sensor is ~0.884 nm/°C.

**Figure 2:**
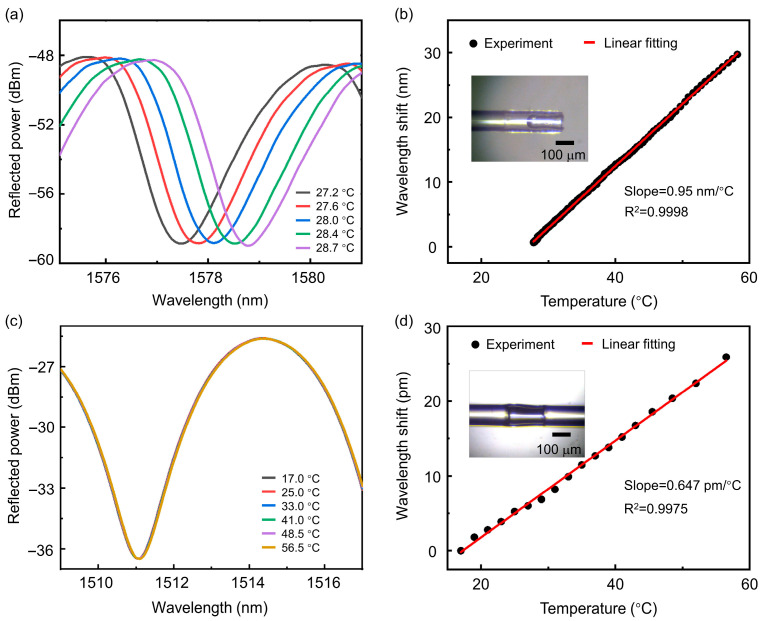
Characterization of the temperature sensing performance of different FPI sensors: (**a**) Monitoring the spectral shift in the PFPI sensor at different temperatures. (**b**) The linear resonant wavelength shift in PFPI related to temperature. The inset is a microscopic image of a PFPI sensor. (**c**) Monitoring the spectral shift in the air-filled Fabry–Pérot interferometer (AFPI) sensor at different temperatures. (**d**) The linear resonant wavelength shift in the AFPI sensor related to temperature. The inset is a microscopic image of an AFPI sensor.

To determine the temperature characteristics of the sensor, a ceramic heating plate (SENXTE, 40 × 40 mm) was used as the heat source, and the temperature of the ceramic heating plate was controlled by adjusting the input electrical current. The sensor under test and a commercial thermocouple sensor were placed together on the surface of the ceramic heating plate, and the input current was gradually increased to raise the temperature slowly. The optical fiber demodulator was used to record the spectral shift, and a digital recorder monitored the real-time temperature change from the thermocouple. After processing the temperature change data and wavelength shift data, the temperature response curve of the sensor was obtained. Figure 2a shows the typical spectral shift in the PFPI sensor correlated to external temperature. The extracted temperature sensitivity is as high as 0.95 nm/°C (Figure 2b), slightly larger than the theoretical calculation. Notably, the ultrahigh temperature sensitivity of PFPI sensors is two orders of magnitude larger than that of commercial FBG sensors. The difference between the measured results and the calculated value is probably attributed to the irregular expansion of the PDMS surface. As shown in Figure 2b inset, the PDMS-air interface formed during the curing process exhibits a characteristic concave morphology, with the central region depressed relative to the periphery. This non-planar geometry creates an enhanced optical path length change. As a comparison, the temperature sensing performance of a conventional AFPI sensor is shown in Figure 2c,d. The extracted temperature sensitivity is only 0.647 pm/°C, three orders of magnitude smaller than that of PFPI sensors.

### 2.2. Cross-Correlation Algorithm for Wide-Range Spectral Shift Tracing

While achieving a remarkable enhancement in temperature sensitivity, we identify a new challenge arising from the fundamental characteristics of the PFPI spectrum. The quasi-sinusoidal spectral output exhibits periodic behavior manageable in conventional systems, where temperature-induced shifts remain within half of the spectral FSR. In such cases, we can reliably track temperature changes by monitoring a single reference peak (typically the extremum of one period) through conventional smoothing and fitting algorithms, as its displacement remained confined to predictable wavelength bounds. However, the dramatically improved sensitivity of our new sensor introduces a critical limitation: substantial temperature variations now frequently exceed one complete FSR. This leads to ambiguous peak identification, where (1) the original reference peak may shift by tens of nanometers, and (2) adjacent periodic peaks may occupy the original reference wavelength position. Such aliasing effects create fundamental measurement uncertainties when using conventional peak-tracking methods.

**Figure 3:**
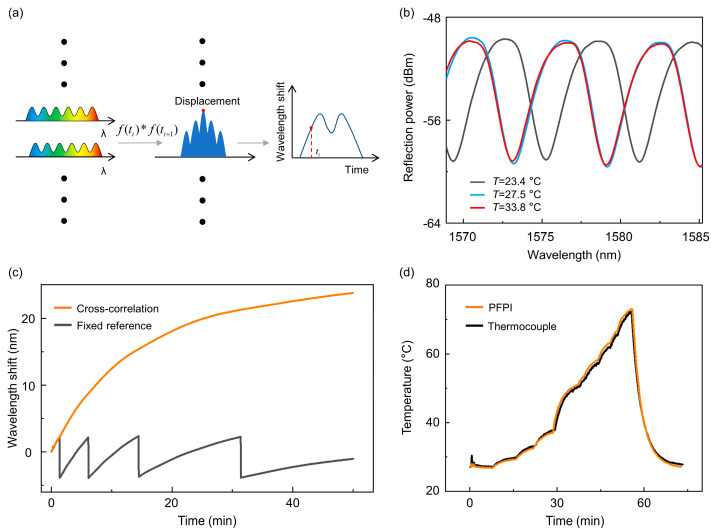
Cross-correlation algorithm for wide spectral shift tracking: (**a**) The schematic of the cross-correlation algorithm for spectral shift tracking. “✱” denotes the cross-correlation operation of the two. (**b**) Typical spectra of the PFPI sensor at different external temperatures. (**c**) Monitoring the wavelength shift over a large dynamic range using the fixed reference method and the cross-correlation algorithm, respectively. The fixed reference method makes incorrect judgments when the wavelength shift spans free spectral range, while the cross-correlation algorithm can achieve stable wavelength tracking results. (**d**) Typical results of monitoring temperature change in a ceramic heating plate using a PFPI sensor and thermocouple.

To solve the spectral track ambiguity issue, we implement a cross-correlation algorithm that quantitatively analyzes the global similarity between interferometric spectra. The discrete cross-correlation function is defined as:

(3) $$R_{xy}\left(p\right)=\sum_{q=-\infty }^{\infty }x[q]y[q+p]$$

where *x*[*q*] and *y*[*q*] represent two different sets of data for cross-correlation calculations, and *p* denotes the delay. A schematic diagram of the cross-correlation calculations is shown in Figure 3a. In the periodically collected spectral data, a spectral window of appropriate bandwidth is selected, and cross-correlation is performed between adjacent spectra to determine the wavelength shift corresponding to temperature variations over time. The experimental light source is provided by the internal laser of the optical fiber demodulator (TV130, Tongwei Sensing, Chengdu, China), and the light source power is 100 μW, covering the spectral range of 1500–1600 nm. The spectral acquisition speed of the system is tunable (1~100 Hz), and the spectral sampling interval is 5 pm. In different operational environments—particularly those with varying rates of temperature change—each spectral set can be cross-correlated with multiple adjacent spectra. A weighted average of these correlations can then be computed to improve the accuracy of the temperature estimation.

Figure 3b shows typical spectra of a PFPI sensor under temperature changes of 4.1 °C and 10.4 °C, respectively. The spectral shifts exceed an FSR, and thus, the conventional fixed reference method cannot correctly retrieve the actual spectral shift. Figure 3c compares the wavelength tracking results using the fixed reference method and the cross-correlation method. It is evident that each time the wavelength change surpasses an FSR, the fixed reference method fails to track the wavelength correctly, resulting in misinterpretations. To evaluate the effectiveness of our temperature tracking algorithm under large-scale temperature variations, we created a test environment using ceramic heating plates. The temperature of the ceramic heater was controlled via a power supply, and both the PFPI sensor and a commercial thermocouple were placed on its surface. Figure 3d shows the response curve of the PFPI sensor and the thermocouple. By tracing the spectral shift in PFPI sensors using the cross-correlation algorithm and knowing the initial ambient temperature, the unknown temperature of objects can be accurately measured based on the measured temperature sensitivity of PFPI sensors (Figure 2b). The close agreement between the two demonstrates the accuracy and applicability of our temperature tracking algorithm for the PFPI sensor.

## 3. Results

### 3.1. Scanning Thermal Field Imaging

**Figure 4:**
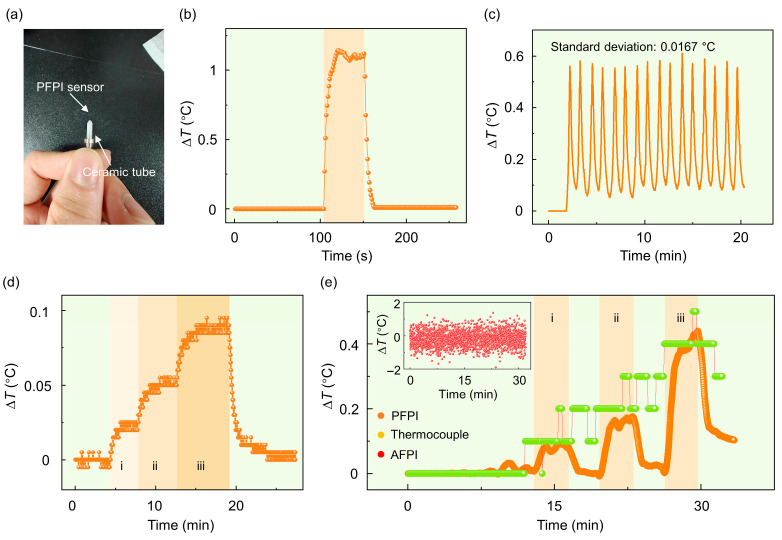
Dynamic response and temperature resolution ability of the PFPI sensor: (**a**) Camera image of the PFPI sensor installed in a ceramic tube. The PFPI sensor is protruding from the ceramic tube. (**b)** Response speed measurement of a PFPI sensor. (**c**) Repeated temperature measurement of an energized resistance wire using a PFPI sensor. (**d**) Using an energized resistance wire as a heat source to test the temperature-resolution ability of the PFPI sensor. Regions i–iii indicate the input electric currents of 0.3 A, 0.35 A, and 0.4 A. (**e**) Comparing the temperature sensing capabilities of the PFPI, thermocouple, and AFPI sensors. Regions i–iii indicate the inlet electric current of 0.4 A, 0.5 A, and 0.6 A. The inset is the temperature response of an AFPI sensor, and the temperature change in the test system cannot be resolved.

In this section, we explore the application of PFPI sensors for scanning thermal field imaging. Firstly, we evaluate the dynamic response and temperature resolution capabilities of the PFPI sensors, as shown in Figure 4. In the experiment, a thermally energized thermal resistance wire (galvanized iron wires, 1 mm in diameter) is used as a localized heat source. By modulating the input current, we control the distribution of the thermal field. The entire experiment is conducted in a relatively closed environment, isolated by an acrylic hood to minimize the impact of external air flow disturbance. To enhance the mechanical stability of the PFPI sensors, we install the sensors in a ceramic tube, as shown in Figure 4a. Notably, the PFPI probe protrudes slightly from the end of a ceramic tube to reduce the background heat capacity and thereby optimize the dynamic response.

Figure 4b shows the dynamic response of the PFPI sensor, demonstrating its ability to respond rapidly to temperature changes. The response time from the beginning of temperature change to stability is approximately 14 s. The response time can be further improved by optimizing the heat capacity of the supporting ceramic tube. Repeatability of sensor response after performing a rapid 16-repeatability test, the standard deviation was only 0.0167 °C, and the consistent results of the sensor were confirmed, as shown in Figure 4c. To assess the temperature resolution of the PFPI sensor, electric currents of 0.3 A, 0.35 A, and 0.4 A were sequentially applied. The corresponding measurements clearly exhibit a stepwise response with a high signal-to-noise ratio, resolving temperature changes as small as 0.025 °C (Figure 4d). Notably, given the sampling interval (5 pm) of the optical interrogator, the ultimate resolution of the PFPI sensor is ~5.3 × 10^−3^ °C. A comparative analysis of PFPI, AFPI, and commercial thermocouple sensors is presented in Figure 4e. The PFPI sensor consistently exhibits stable performance and promptly returns to its initial state following each cessation of current input. In contrast, the thermocouple sensor, with a detection limit of 0.1 °C, displays lower accuracy and delayed temporal response. For the AFPI sensor, due to its very low temperature sensitivity of 0.647 pm/°C, the wavelength shift under a temperature change of 0.4 °C remains below the 5 pm sampling interval of the interrogator. As a result, the temperature signal becomes indistinguishable from system noise. Notably, limited by the response time of PFPI sensors, pure-silica fiber-optic sensors (Figure 2d) are more appropriate for scenarios involving rapid temperature fluctuations. Nevertheless, we contend that the proposed PFPI sensor remains well-suited for highly sensitive temperature measurements in most everyday environments.

**Figure 5:**
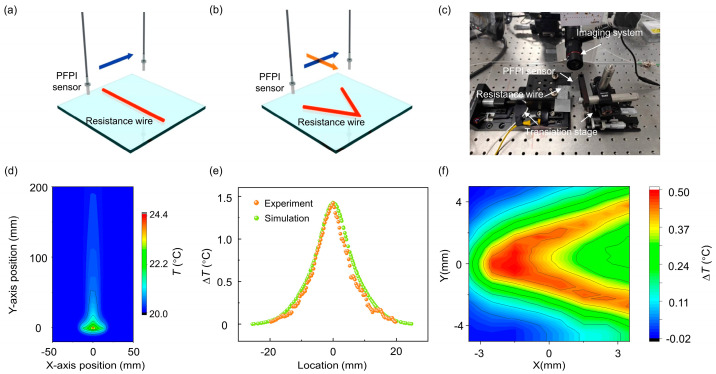
Scanning thermal field imaging using the PFPI sensor: (**a**) Schematic diagram of one-dimensional (1D) thermal field reconstruction, using a straight energized resistance wire as the heat source. (**b**) Schematic diagram of two-dimensional (2D) thermal field reconstruction, using a V-shaped energized resistance wire as the heat source. The thermal field reconstruction process requires the use of a multi-axis translation stage for mechanical scanning. (**c**) Camera image of the experimental setup. The imaging system is used to determine the relative position between the sensor and the heat source. (**d**) Simulation of the thermal field distribution of an energized resistance wire. (**e**) Comparison between the measured 1D temperature distribution and the simulation results. The PFPI sensor is placed at a height of 1 cm above the resistance wire. (**f**) The measured 2D thermal field distribution of the V-shaped wire.

A thermal field reconstruction experiment was subsequently carried out. Figure 5a,b show the schematic diagrams of the heat sources used to generate one-dimensional (1D) and two-dimensional (2D) thermal fields, respectively. The heat sources are constructed using electrified resistance wires. By scanning the PFPI sensor in a single direction above the wire using a translation stage, the 1D temperature distribution is obtained. In the 2D case, a V-shaped resistance wire was used to construct a spatially varying thermal field. The sensor was swept along two orthogonal directions to map the temperature distribution of the corresponding plane. The experimental setup is depicted in Figure 5c. The PFPI sensor, enclosed in a ceramic tube, was mounted on an optical translation stage, serving as the sensing probe. The resistance wire was fixed on another translation stage, functioning as the thermal field generator. The current through the resistive wire is controlled by using a DC power supply, so that the temperature of the resistance wire gradually rises to a steady state. A microscopic imaging system was positioned laterally to monitor the relative position between the PFPI sensor and wire, facilitating precise distance adjustments. The entire setup was enclosed in a custom acrylic cover to minimize temperature interference from ambient airflow.

A simulation tool was employed to simulate the heat generation of the resistance wire and model the thermal field. Since the sensor and heat source are physically separated, only heat convection and thermal radiation were considered—thermal conduction was negligible. The simulation results, shown in Figure 5d, reveal that heat from the energized wire causes a temperature rise in the surroundings, with upward airflow and convective heat transfer. Simulated temperature data at a plane 1 cm above the resistance wire were extracted and compared with experimental measurements. As shown in Figure 5e, the two sets of data exhibit excellent agreement, validating the feasibility of using the PFPI sensor for 1D thermal field reconstruction. The 2D thermal field map is shown in Figure 5f. A clear temperature distribution was obtained across a planar area of 7 × 10 mm^2^, demonstrating the PFPI sensor’s potential for high-resolution micro-scale thermal field sensing.

### 3.2. Body Temperature and Respiratory Signals Monitoring

**Figure 6:**
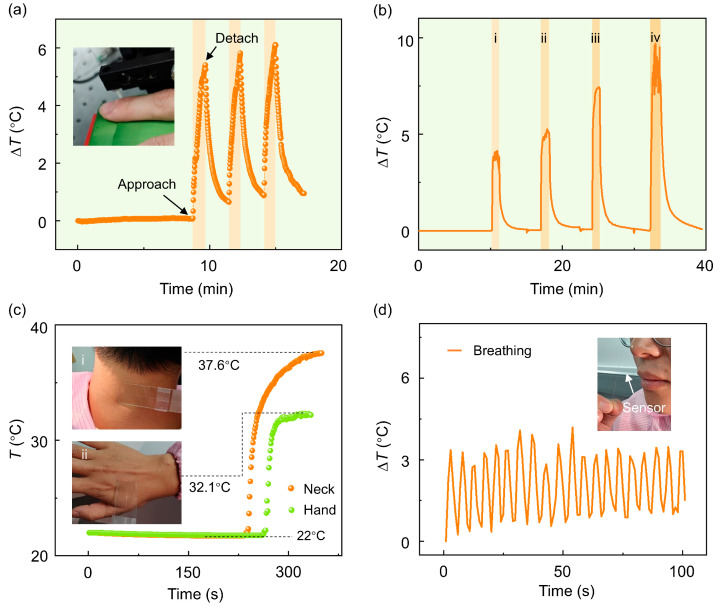
Monitoring of human body temperature and respiratory signals by using the PFPI sensor: (**a**) Non-contact sensing performance of the PFPI sensor. A finger was positioned 3 mm below the sensor, and temperature variations induced by human thermal radiation were successfully detected, with repeatable results across trials. (**b**) Non-contact sensing performance of the PFPI sensor at different distances. Regions of i–iii correspond to distances of 13 mm, 8 mm, and 3 mm, respectively, and region iv presents results of contact measurement. (**c**) The measured results of the PFPI sensor for contacting different locations of the human body. (**d**) The measured results of the PFPI sensor for respiratory signal monitoring. The insets in (**a**,**c**,**d**) present real-scene camera views captured during the sensor measurements.

We subsequently explore the use of the PFPI sensor for non-contact temperature sensing, human body temperature monitoring, and respiratory signal detection. Figure 6a presents the results of non-contact finger temperature sensing using the PFPI sensor. The optical interrogator is configured to record data at a rate of 1 Hz. Prior to 520 s, the system remained in a stationary state, during which a slight increase in ambient temperature was observed. After that, a finger was moved into the detection area. The relative height between the sensor and the base of the detection area was pre-calibrated to maintain a fixed sensor-to-finger distance of approximately 3 mm. A steadily rising sensing signal was recorded during the presence of the finger. After 60 s, the finger was withdrawn, resulting in a continuous decrease in the detected temperature signal. This process was repeated three times, and similar waveforms were obtained across all trials, demonstrating the reproducibility and potential of the PFPI sensor for non-contact human temperature monitoring. Figure 6b shows the results of the sensor detection signals at different distances. For regions i–iii, the finger is at distances of 13 mm, 8 mm, and 3 mm from the sensor, respectively. In particular, region iv shows the sensing result when the finger directly contacts the sensor. At this time, the sensor signal fluctuates greatly, which may be related to the shaking of the finger.

The PFPI sensor was further evaluated for its potential application as a human body thermometer. In this experiment, the sensor was affixed to a glass slide to avoid possible collision damage, and the reverse side of the slide was brought into direct contact with different parts of the body. Figure 6c presents the measured temperature signals from the back of the hand and the neck. The recorded temperatures were approximately 32.1 °C and 37.6 °C, respectively, consistent with typical human body temperature distributions. Due to the relatively large size and thickness of the glass slide, a longer duration was required to reach thermal equilibrium. For example, the time stabilization for hand temperature measurement is approximately 28 s. In future research, seeking proper encapsulation materials and structures will be in demand for practical applications. Figure 6d shows the results of respiratory monitoring using the PFPI sensor. The sensor was positioned near the philtrum while the volunteer performed nasal breathing. The thermal airflow produced by exhalation led to periodic temperature variations. Although the current sampling rate limited the number of data points captured within each breathing cycle, the sensor still clearly exhibited a periodic signal, demonstrating the feasibility of breath signal detection. With the implementation of a higher-speed data acquisition system, the PFPI sensor could potentially provide more detailed respiratory metrics, such as breathing rate, depth, and pattern quality. This suggests a promising optical sensing approach for monitoring human respiratory health.

## 4. Discussion

In summary, we demonstrate the self-adaptive PFPI sensors for ultrasensitive and wide-linear-range thermal field detection. The developed PFPI sensors show a temperature sensitivity of 0.95 nm/°C, which is two orders of magnitude larger than that of the conventional FBG sensors. The PFPI sensors also have higher temperature sensitivity and faster response recovery than commercial thermocouple sensors. The use of PFPI sensors for micro-region thermal field detection shows that the sensor has high temperature resolution and has a promising future in microscopic temperature sensing applications. In order to explore more practical application scenarios, we try to apply the sensor to human body temperature monitoring as well as respiratory signal monitoring, which shows the potential of PFPI sensors in the field of human health monitoring.

## Data Availability

Data are not publicly available at this time but may be obtained from the authors upon reasonable request.

## Abbreviations

The following abbreviations are used in this manuscript:

FBG	fiber Bragg grating
PDMS	polydimethylsiloxane
FPI	Fabry–Pérot interferometer
APFI	air-filled Fabry–Pérot interferometer
FSR	free spectral range
